# No molecular evidence of Borna disease virus among schizophrenia and bipolar disorder patients in Iran

**Published:** 2017-04

**Authors:** Somayeh Shatizadeh-Malekshahi, Hamid Reza Ahmadkhaniha, Seyed Jalal Kiani, Ahmad Nejati, Leila Janani, Jila Yavarian

**Affiliations:** 1 Virology Department, School of Public Health, Tehran University of Medical Sciences, Tehran, Iran; 2 Mental Health Research Center, Department of Psychiatry, Iran Mental Hospital, Iran University of Medical Sciences, Tehran, Iran; 3 Department of Biostatistics, School of Public Health, Iran University of Medical Sciences, Tehran, Iran

**Keywords:** Bipolar disorder, Borna disease virus, Iran, Nested RT-PCR, Schizophrenia

## Abstract

**Background and Objectives:**

Viruses have been suggested as one of the risk factors for psychiatric disorders. Among infectious agents Borna disease virus (BDV) has been known as a neurotropic virus which is able to cause neurological disorders in different animals. Recently there were controversial findings about BDV association with pathogenesis of human psychotic disorders.

**Materials and Methods:**

Here we performed a nested reverse transcription polymerase chain reaction for detection of BDV P40 RNA in peripheral blood mononuclear cell samples of schizophrenia (SC), bipolar disorder (BD) patients and healthy controls (HCs).

**Results:**

Only one out of 120 (0.8 %) psychiatric patients and two samples (2.7%) in 75 HCs showed positive results. There were no significant molecular evidence of BDV infection in 120 psychotic patients (60 SC and 60 BD) and 75 matched HCs.

**Conclusion:**

Our findings showed no association between BDV infection and pathogenesis of these psychiatric disorders. This is an interesting issue given both the as yet un-clarified role of BDV in human mental disorders and addressing patients in the so far under-investigating Middle East era.

## INTRODUCTION

Borna disease virus (BDV) has been recognized as a causative agent of meningoencephalitis in horses, sheep and several vertebrate species at the end of 19 ^th^ century in Germany (1). Depending on the animals, the virus infection can induce various behavioral and neurological abnormalities (2). Borna disease virus is classified in the family *bornaviridae* within the order of *mononegavirales* and has non-segmented, negative sense RNA genome of approximately 9 kb which contains 6 open reading frames (ORFs) (3).

According to the epidemiological and molecular profile of BDV in Europe, Asia and USA a picture emerges that it could also infect the humans (4). Other investigators have suggested the possible relationship between BDV and human psychiatric diseases in various regions such as Europe, Brazil and Japan (5–7). Additionally, seroepidemiological data and detection of BDV RNA in peripheral blood mononuclear cells (PBMCs) provide a possible involvement of BDV in human psychiatric disorders (8–10). However, there is much dispute in the field regarding the prevalence of BDV antibodies and RNA in the PBMCs of patients with psychiatric disorders (11–12). Psychiatric disorders like schizophrenia (SC) and bipolar disease (BD) have been affecting general population and their etiology remains unknown despite several decades of intensive research (13). Apart from gaining attention as a causative agent in psychiatric disease, BDV has recently been identified to enter the genome and endogenous bornalike N (EBLN) elements homologous to the BDV nucleoprotein (N) gene exist in the genome of several mammalian species (14–15). A novel Bornavirus, which causes human disease (fatal encephalitis), is the zoonotically transmitted variegated squirrel 1 bornavirus (VSBV-1) (16).

In the present study, we have investigated the prevalence of BDV in the PBMCs of patients with BD, SC and in healthy controls (HC) by nested reverse transcriptase PCR (RT-PCR) for the amplification of a fragment of ORF-I; coding for p40 nucleoprotein.

## METHODS

### Subjects.

Patients with DSM-IV diagnosis (17) of BD and SC who were hospitalized between March and September 2013 in Iran Mental Hospital located in Tehran were enrolled in this study. All were chronically ill which receiving antipsychotic medications at the time of this research. Patients with intravenous drug abuse and substance use disorders were excluded. This research was approved by the Ethics Committee of Tehran University of Medical Sciences and informed consent was filled in with all patients after full description of the study.

For HCs recruitment, there were some options as follow:

1. Patient’s relatives: due to the strong association between genetic factors and mental illness and possible transmission of the virus it was problematic.
2. Blood donors: it wasn’t possible because fresh blood was mandatory and taking such samples were difficult.
3. People who lived very close to patient’s residential area: convincing the people for taking blood wasn’t easy to perform.

In this regard HCs were selected from university staff according to SCID (Structured Clinical Interview for DSM Disorders) (18) with no history of mental disorder, no hospital admission and no relationship (relative, household or sexual partner) with the case subjects. Socioeconomic status, geographic region and sex were the same between control and case groups with similar age (± 2 years).

### Preparation of peripheral blood mononuclear cells (PBMCs).

A total of 10 ml whole blood samples were collected by venipuncture from each subject in the presence of anticoagulant EDTA and RNAse free tubes. PBMCs were separated using Ficoll (Ficoll-paque ^TM^ -plus) a density gradient medium on the day of blood drawing according to the established protocol (19). PBMCs were washed twice with phosphate buffer saline (PBS) and finally resuspended in heat-inactivated fetal calf serum (FCS) with 10% DMSO (Dimethyl sulfoxide), progressively cooled down to −80°C and stored in liquid nitrogen until use.

### RNA extraction.

For RNA extraction, cryopre-served PBMCs were rapidly thawed in a water bath at 37°C and washed twice with PBS. Total RNA was extracted from PBMCs using the viral RNA extraction kit (Roche, Germany) according to the manufacturers instruction. The approximate concentration of extracted RNA was assessed by optical density (OD) at 260/280 ratios.

### Detection of BDV RNA in PBMCs by nested RT-PCR.

The *in vitro* synthesized PUC57 plasmid containing desired fragment of BDV p40 refseq was used as the positive control and aliquots which contain all reagents except the target sequence was utilized as negative controls in each run. Borna disease virus P40 could be good target for the diagnosis of BDV infection because recent studies demonstrated that this protein play important roles in virus replication and transcription (20). To prevent contamination, RNA samples and the PCR master mixes were prepared in a biosafety hood in different rooms. PBMC RNA was screened for the presence of BDV p40 by nested RT-PCR. cDNA synthesis was carried out in 30 μl reaction mixture containing 6 μl of 5X RT Buffer, 2.5 μl of mixed dNTPs (2.5μm each), 1 μl of RT-MULV enzyme (Thermo Fisher scientific), 2.5 μl Random hexamer (Thermo Fisher scientific), 0.5 μl RNase inhibitor (Thermo Fisher scientific) and 17.5 μl RNA template, the mixture was incubated at 22°C for 10 min, 37°C for 45 min and 94°C for 5 min. After reverse transcription, the PCR was performed in a 50 μl reaction mixture. For the first round of PCR, 10 μl of the cDNA product and for the second round, 5 μl of the first-round PCR product was used. Primer pairs used for the first and second rounds of BDV p40 PCR are indicated in Table 1 (10). Each PCR contained 0.2 mM of each primer, 200 mM dNTPs, 1.5 mM MgCl _2_ , 10 mM Tris-HCl (pH 8.3), 50 mM KCl, and 0.5 U of Taq polymerase in a final volume of 50. The conditions of the first and second PCRs were as follows: pre-denaturation at 94°C for 1 min followed by 40 cycles of 94°C for 1 min, 55°C for 1 min, 72°C for 1 min and a final extension of 10 min at 72°C. The PCR products were separated by 1% agarose gel electrophoresis. To confirm the presence of BDV RNA, RT-PCR was duplicated for all of the samples. For evaluation of the sensitivity of nested RT-PCR for BDV p40, after serial dilutions of plasmid containing the BDV p40 cDNA (pUC57), positive control was used in each RT-PCR assay.

**Table 1.** The sequences of primers used for nested RT PCR amplification of BDV P40.

**Primers**	**Sequences**	**Nucleotide position**
	First round	
Sense	5′-TTCATACAGTAACGCCCAGC-3′	259–278
Antisense	5′-GCAACTACAGGGATTGTAAGGG-3′	808–829
	Second round	
Sense	5′GCCTTGTGTTTCTATGTTTGC-3′	277–297
Antisense	5′GCATCCATACATTCTGCGAG-3′	786–805

### Sequencing of PCR products.

Positive PCR products were purified by QIAquick PCR Purification Kit (Qiagen). The purified products were then sequenced bidirectionally with inner primers specific for P40 region, using a BigDye terminator cycle sequencing kit (Applied Biosystems) and a 3,130 genetic analyzer (Applied Biosystems). Sequences were analyzed by GenBank BLASTn nucleotide search and submitted under GRP4432276-78 accession numbers.

### RNA quality control.

To evaluate the quality of the RNA isolated from PBMCs, RT-PCR for a house keeping gene, β-Globin mRNA with sense primer 5′-ACACAACTGTGTTCACTAGC-3- and antisense primer 5′-CAACTTCATCCACGTTCACC-3′ was carried out to correct variation of all samples. The protocol will be available upon request.

### Statistical analysis.

Continuous variables are presented as mean (SD) and qualitative variables are reported through frequencies (percentages). The normality of continuous variables was assessed using graphical methods and also Shapiro-Wilk test. Because the distribution of variables was not markedly skewed, the two-sided Independent t test was used to compare the mean of continuous variables between cases and controls. Distribution of qualitative variables was compared through Chi-squared test and Fisher’s exact test (for tables when more than 20% of expected frequencies are lower than 5). Statistical analysis was performed using SPSS 18.0 software (SPSS Inc., Chicago, IL, US). P-values less than 0.05 were considered statistically significant.

## RESULTS

### Demographical characteristics.

The study samples were consisted of 120 patients (60 with BD and 60 with SC) and 75 HCs. Among patients 78 (65%) were male and 42 (35%) were female the same as matched control groups (64% male; 36% female). The proportion of males was higher in SC (76.7%) than BD patients (53.3%). Mean age of the patients was not different from the HCs. In 60 SC patients there was no difference in season of birth (25% was born in each season) but in BD patients 35% were born in winter, 25% in spring, 25% in summer and 15% in autumn. In BD and SC patients 44% & 29% had family medical history of these disorders, respectively. Socioeconomic status, ethnicity and country of origin were similar across the groups. Some characteristics of the subjects are summarized in Table 2.

**Table 2.** Subject Characteristic

	**Cases**			**Controls** (N=75)	**P-value**

	**Schizophrenia** (N=60)	**Bipolar disease** (N=60)	**Total** (N=120)		
**Age**					
Mean (SD)	35.95 (9.99)	35.87 (11.58)	35.91 (10.77)	35.59 (10.74)	0.839^a^
**Sex**					
Male (%)	46 (76.7%)	32 (53.3%)	78 (65.0%)	48 (64.0%)	0.887^b^
Female (%)	14 (23.3%)	28 (46.7%)	42 (35.0%)	27 (36.0%)	
**Marital status**					
Single (%)	41 (68.3%)	31 (52.5%)	72 (60.5%)	35 (48.5%)	0.090^c^
Married (%)	17 (28.3%)	27 (45.8%)	44 (37.0%)	37 (51.4%)	
Divorced (%)	2 (3.4%)	1 (1.7%)	3 (2.5%)	0 (0.0%)	
**Family history**					
Yes (%)	18 (30.0%)	26 (43.3%)	44 (36.7%)	8 (10.7%)	
No (%)	42 (70.0%)	34 (56.7%)	76 (63.3%)	67 (89.3%)	
**Hospital admission**					
**history**					
Yes (%)	58 (96.7%)	56 (93.4%)	114 (95%)		
No (%)	2 (3.3%)	4 (6.6%)	6(5%)		
**Smoking**					
Yes (%)	31 (61.7%)	14 (23.3%)	45 (37.5%)		<0.001^b^
No (%)	29 (48.3%)	46 (76.7%)	75 (62.5%)		

Notes: SD = Standard deviation, a = Significances are based on t-test, b = Significances are based on Chi-square test, c = significances are based on Fisher-exact test.

### RNA quality control.

**Fig. 1.**
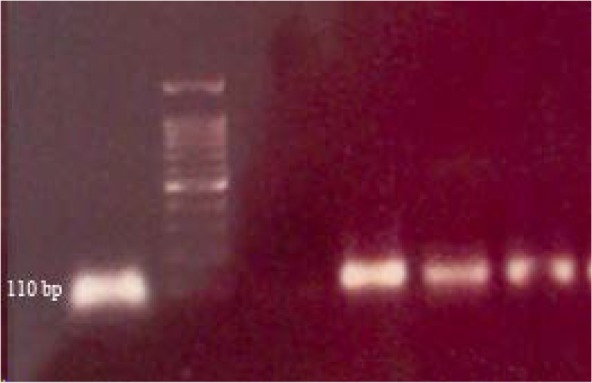
β-Globin RNA analysis by agarose gel electrophoresis.

The result of RNA quality control of β-Globin mRNA was shown in Fig. 1.

### Detection of BDV p40 RNA in PBMCs of psychiatric patients and HCs by nested RT-PCR.

To investigate the prevalence of BDV RNA, nested RT-PCR was used to test the RNA extracted from the PBMCs. The RT-PCR assay specifically yielded a predicted 528-bp DNA fragment. Only one out of 120 (0.8 %) psychiatric patients and two samples (2.7%) in 75 HCs showed positive results. The only positive SC sample was born in winter with family history of this disease.

### Sequencing of BDV RT-PCR products & homology searches.

The validity of the positive products was confirmed by sequencing, which revealed a greater than 96 % identity with BDV p40 fragments in GenBank. In addition no common mutation in samples was found.

## DISCUSSION

Borna virus disease is a neuro-infectious disorder in a wide range of animals including cattle, cats, birds and primates. The infection of BDV in the central nervous system (CNS) of animals may cause encephalomyelitis, meningitis and behavioral abnormalities (21). It worth mentioning that in one Japanese study the BDV prevalence was shown 27.8% (20/72) in Iranian horses (22). Besides, the only so far published study which had addressed Iranian psychiatric patients as well found considerable infection rate through serological methods (23). Blood donors and sex- and age matched, mentally healthy subjects were included as controls in that study.

Detection of BDV RNA in PBMCs of experimentally infected neonatal rats (24–25) hold the potential to reveal BDV etiology in human psychiatric disease. Indeed, the wide host range of the virus and behavioral disturbances in animals with BDV suggest that BDV infection may be associated with human psychiatric disorders. Moreover, experimental BDV infection of animals such as rats induces behavioral changes that resemble some types of affective neuropsychiatric disorders in human (25). Recent epidemiological studies have reported controversial results between BDV infection and human psychiatric disorders (11, 26). A very recent study described a novel divergent Borna-virus infecting squirrels with zoonotic potential (and several human deaths), which provides new arguments for BDV human pathogenesis (16). In addition, knowing the status of BDV infections in Iran would be interesting per se.

In previous studies different serological methods have been developed to detect BDV infections (27). In recent years, PCR assay has been used as a promising method for the diagnosis of different neurotropic virus infections (4). In this study, we have successfully performed a reliable method to detect BDV p40 RNA from PBMCs by using nested RT-PCR. This case-control study by analyzing 120 patients with SC and BD disorders and 75 HCs did not reveal evidence of BDV association with these disorders using molecular assay.

In prior studies, there has been long history of controversial results in the prevalence of BDV RNA in the PBMCs of psychiatric patients. Sauder et al (10) reported a high prevalence (38.5%) of BDV RNA in psychiatric patients in Homburg, Germany. Kishi et al (25) also showed a high prevalence (37%) of BDV RNA in Japanese psychiatric patients compared with HCs (6.5%). Overall, there are many more references reporting either positive result for RNA in human PBMCs or correlative data of virus infection and disease, which are based on RNA and serology (28–29). Nonetheless another study in Japan was reported a low prevalence (1.9%) of BDV p24 RNA in psychiatric patients and no viral RNA in HCs (30). Richt et al. (31) and Lieb et al. (32) reported the absence of BDV RNA in PBMCs of either psychiatric patients or HCs. Na et al. (33) in Korea and Horing et al. (34) failed to detect BDV RNA in psychotic patients. This might be due to the geographic differences between the groups with respect to clinical course, diagnosis or differential exposure to infectious organisms (10, 34). However, we should consider the variation in sample size, subject recruitment, sampling procedures, collection and processing as an explanation for differences obtained between studies. Moreover, methodological disparities due to different antibody or RNA techniques lead to great variation in prevalence results. Dürrwald et al. (26) in a meta-analysis study suggested that the studies with BDV-positive results by nested RT-PCR might have used contaminated samples. Because nested RT-PCR is prone to contamination we particularly paid attention to keep all the equipments cleaned and decontaminated. However, several studies have shown positive RT-PCR results were higher in patients than in controls (10, 35). In addition, it has been suggested that BDV infection in humans would be asymptomatic and non-pathogenic (36) then, it can be concluded that the symptoms and pathogenesis of BDV infection in humans would be different from the infection in animals (33).

Recent studies on the incorporation of BDV sequences into the genome of human and other mammals have suggested that primates were infected by bornaviruses over 40 million years ago (15). This amazing finding argues strongly in favor of human BDV infection as a potential hazard.

Analyzing the sequences of positive specimens represented a high degree of conservation with BDV p40 fragments submitted in GenBank. RNA viruses show high mutation rate because of error-prone replicase, though can also reveal long lasting stasis. There are hypothetical options regarding this high sequence conservation. In this context an unidentified proofreading function of BDV polymerase or results of selective pressure can be considered (10, 37).

In summary, we investigated an interesting and neglected infection with potential impact on psychiatric disorders. Because nested RT-PCR is less sensitive than real time RT-PCR negative results may not exclude infection and this limitation should be considered in the future studies. The reported findings are in line with those groups which find no association of BDV with psychiatric diseases. Absence of evidence does not virtually equal to something that does not exist. Nevertheless, our results do not support the hypothesis that BDV may be implicated in the pathogenesis of SC and BD patients.

In conclusion, we propose to perform a comprehensive study of BDV infection over longer period of time with the larger sample size. The findings might be valuable for a better understanding of the etiology and prevalence of BDV infection in psychiatric disorders. This is an interesting issue given both the as yet un-clarified role of BDV in human mental disorders and addressing patients in the so far under-investigating Middle East era.

## Abbreviation:

BDV: Borna disease virus
BD: Bipolar disorder
CNS: Central nervous system
DSM-IV: Diagnostic and Statistical Manual of Mental Disorders, Fourth Edition
HC: Healthy control
PBMC: Peripheral blood mononuclear cell
PCR: Polymerase chain reaction
SC: Schizophrenia
SCID-I: Structured Clinical Interview for DSM-IV Axis I Disorders.
